# Risk stratification in GIST: shape quantification with CT is a predictive factor

**DOI:** 10.1007/s00330-019-06561-6

**Published:** 2020-01-03

**Authors:** Sheng-cai Wei, Liang Xu, Wan-hu Li, Yun Li, Shou-fang Guo, Xiao-rong Sun, Wen-wu Li

**Affiliations:** 1 Department of Nuclear Medicine, Shandong Cancer Hospital and Institute, Shandong First Medical University and Shandong Academy of Medical Sciences, No 440 Jiyan Road, Jinan, 250117 Shandong Province People’s Republic of China; 2 Department of Radiology, Shandong Cancer Hospital and Institute, Shandong First Medical University and Shandong Academy of Medical Sciences, No 440 Jiyan Road, Jinan, 250117 Shandong Province People’s Republic of China

**Keywords:** Gastrointestinal stromal tumor, Risk, Tomography, x-ray, Form

## Abstract

**Background:**

Tumor shape is strongly associated with some tumor’s genomic subtypes and patient outcomes. Our purpose is to find the relationship between risk stratification and the shape of GISTs.

**Methods:**

A total of 101 patients with primary GISTs were confirmed by pathology and immunohistochemistry and underwent enhanced CT examination. All lesions’ pathologic sizes were 1 to 10 cm. Points A and B were the extremities of the longest diameter (LD) of the tumor and points C and D the extremities of the small axis, which was the longest diameter perpendicular to AB. The four angles of the quadrangle ABCD were measured and each angle named by its summit (A, B, C, D). For regular lesions, we took angles A and B as big angle (BiA) and small angle (SmA). For irregular lesions, we compared A/B ratio and D/C ratio and selected the larger ratio for analysis. The chi-square test, *t* test, ROC analysis, and hierarchical or binary logistic regression analysis were used to analyze the data.

**Results:**

The BiA/SmA ratio was an independent predictor for risk level of GISTs (*p* = 0.019). With threshold of BiA at 90.5°, BiA/SmA ratio at 1.35 and LD at 6.15 cm, the sensitivities for high-risk GISTs were 82.4%, 85.3%, and 83.8%, respectively; the specificities were 87.1%, 71%, and 77.4%, respectively; and the AUCs were 0.852, 0.818, and 0.844, respectively. LD could not effectively distinguish between intermediate-risk and high-risk GISTs, but BiA could (*p* < 0.05). Shape and Ki-67 were independent predictors of the mitotic value (*p* = 0.036 and *p* < 0.001, respectively), and the accuracy was 87.8%.

**Conclusions:**

Quantifying tumor shape has better predictive efficacy than LD in predicting the risk level and mitotic value of GISTs, especially for high-risk grading and mitotic value > 5/50HPF.

**Key Points:**

*• The BiA/SmA ratio was an independent predictor affecting the risk level of GISTs. LD could not effectively distinguish between intermediate-risk and high-risk GISTs, but BiA could.*

*• Shape and Ki-67 were independent predictors of the mitotic value.*

*• The method for quantifying the tumor shape has better predictive efficacy than LD in predicting the risk level and mitotic value of GISTs.*

## Introduction

Gastrointestinal stromal tumors (GISTs) are the most common mesenchymal tumors. Risk stratification of GISTs tries to evaluate the risk of poor outcome and to choose patients who may benefit from adjuvant therapy [1]. Although criteria may vary from country to country, the 2008 National Institute of Health (NIH) criteria are the most widely used. GISTs are classified into four categories (very low-, low-, intermediate-, and high-risk) according to tumor size, location, mitosis count, and tumor rupture [2]. Tumor size, location, and rupture can be evaluated by CT, whereas mitosis count can only be obtained by pathological evaluation of the surgically removed specimen. Therefore, our primary purpose was to explore CT features that could predict the mitosis value before surgery. Tumor growth pattern or enlarged vessels feeding or draining the mass can help predict the risk [3]. CT features like location, contour, necrosis, enhancement pattern, and tumor calcification are associated with grades [4–6]; few studies report how tumor shape can be correlated with risk grading. In glioblastoma [7] or pancreatic neuroendocrine tumors [8], tumor shape is associated with the outcome. Therefore, we explored a novel method for quantifying the shape of GISTs using CT. In this method, we measured the LD and short diameter (SD) of the lesion and draw four angles A, B, C, and D. The LD corresponds to angle A and angle B (A ≥ B), and SD corresponds to angle C and angle D (D ≥ C). For regular lesions, we take angle A and angle B as BiA and SmA. For irregular lesions, we compare A/B ratio with D/C ratio, select the larger ratio for analysis, and then investigate the relationship between risk stratification and the shape of GISTs.

## Materials and methods

### Patients

**Fig. 1:**
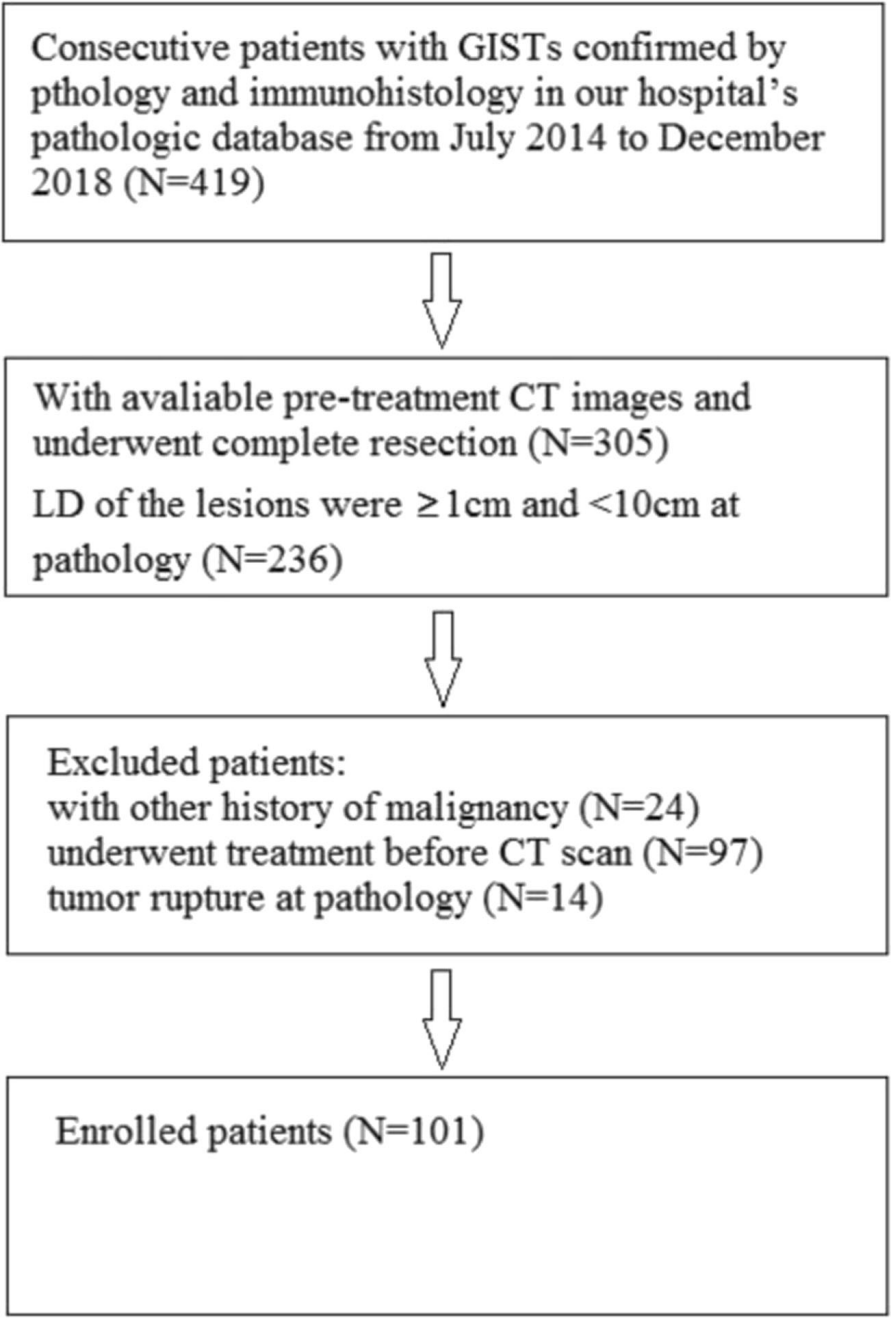
Flowchart showing the inclusion process. GISTs, gastrointestinal stromal tumors; LD, long diameter

We searched the pathological database in our hospital from July 2014 to December 2018 using the search terms “GISTs.” For study inclusion, the following criteria were used: (1) patients diagnosed with GIST by pathology and immunohistology after complete resection (laparoscopic, endo-luminal, or open surgery); (2) patients who had undergone unenhanced and tri-phasic CT before treatment and none of the lesions had tumor rupture at pathology; and (3) the lesion size was ≥ 1 cm and < 10 cm at pathology. The exclusion criteria were as follows: (1) patients with other history of another malignancy; (2) those who underwent any treatment before CT scan; (3) tumor rupture at pathology; and (4) the lesion size was < 1 cm and ≥ 10 cm at pathology. The study workflow diagram with respect to patient selection is shown in Fig. 1. Finally, we enrolled 101 patients (101 lesions in total): 49 males (mean age, 57.4 years ± 11 [standard deviation]; range, 34–85 years) and 52 females (mean age, 57.4 years ± 7.6 [standard deviation]; range, 45–80 years). Among the 101 patients, only one had diffuse growth, and one case had two gastric lesions. In the case of two lesions, one of them grew irregularly and the other one grew regularly (LD = 0.9 cm), and we took the larger one for analysis. This study was approved by the institutional review board (IRB) at the Cancer Center, and informed consent was waived.

Among the 101 patients, 10 were asymptomatic, 38 had abdominal discomfort, 6 had loss of appetite or general fatigue, and 28 had bellyache or distension. In addition, 15 had melena, and 4 had obstructive symptoms such as vomiting. None of the patients underwent any type of therapy before the CT examination.

### Acquisition of CT images

All 101 patients underwent tri-phasic abdominal or abdominopelvic CT scans. All CT scans were obtained using a high-speed 128 or 64 slice spiral CT scanner (Philips Brilliance iCT or Philips Brilliance 64 CT). The scanning parameters were as follows: 120 kV, 250 mAs, 32 × 0.625 collimation, 0.75-s rotation time, 1.7-s cycle time, and 5-mm increments. Each patient consumed 750–1000 mL of tap water or gastrointestinal oral contrast media (Aizhong Medical Imaging) approximately 60 min prior to, and an additional 500-mL contrast immediately preceding CT imaging to maximize both bowel and gastric distension. The contrast medium used was Omnipaque (300 mg/L; General Electric) with 80–100 mL injected via the median cubital vein at a rate of 2.7–3.0 mL/s. For the arterial phase (Ap), a delay time of 25–35 s was used. Venous phase (Vp) and delayed phase (Dp) scanning were performed 60–75 s and 90–120 s after contrast administration, respectively. The images were reconstructed with hybrid iterative reconstruction algorithm (iDose, level 3), standard kernel, contiguous 1-mm-thick slices. We reformatted contiguous 1-mm-thick coronal and sagittal images .

### Image analysis

Two radiologists (LX and WHL) with 10–15 years of experience who were blinded to the pathology results independently reviewed the CT images of GIST on the Picture Archiving and Communication Systems Workstation (PACS). The two radiologists met later to reach a consensus on the results on which they had initially disagreed. The consensus results were used to analyze CT features of GISTs, and the results were from the independent reviews of each. The following CT findings were analyzed: location (stomach, small intestine, or others), contour (regular or irregular), growth pattern [3] (endoluminal, exophytic, or mixed), necrosis, enhancement pattern (homogenous or heterogeneous), and tumor calcification.

Another two radiologists (XRS and WWL) with 15–20 years of experience independently measured and recorded the unenhanced (UE) and tri-phasic CT attenuation coefficient (density) of the tumor in Hounsfield units (HU) by drawing a region of interest (ROI) of the solid components of lesions. The choice of ROI was determined as follows: the same level of ROI was used in each period; necrosis, calcification, hemorrhage, fibrosis, and obvious blood vessels were avoided; and the ROI was at least greater than 30 mm^2^. The CT attenuation coefficients (HU) of the lesion’s same slices in the unenhanced, arterial, venous, and delayed phases were represented by UE, Ap, Vp, and Dp, respectively. Comparing the magnitude of the Ap, Vp, and Dp of the lesion, the phase of the maximum one was used as the enhanced peak period. The absolute enhancement CT attenuation coefficient of the Ap was represented by ApU, and ApU was the subtraction value between the Ap and the UE; the absolute enhancement CT attenuation coefficient of Vp was represented by VpU, and VpU was the subtraction value between the Vp and the UE; the absolute enhancement CT attenuation coefficient of Dp was represented by DpU, and DpU was the subtraction value between the Dp and the UE.

**Fig. 2:**
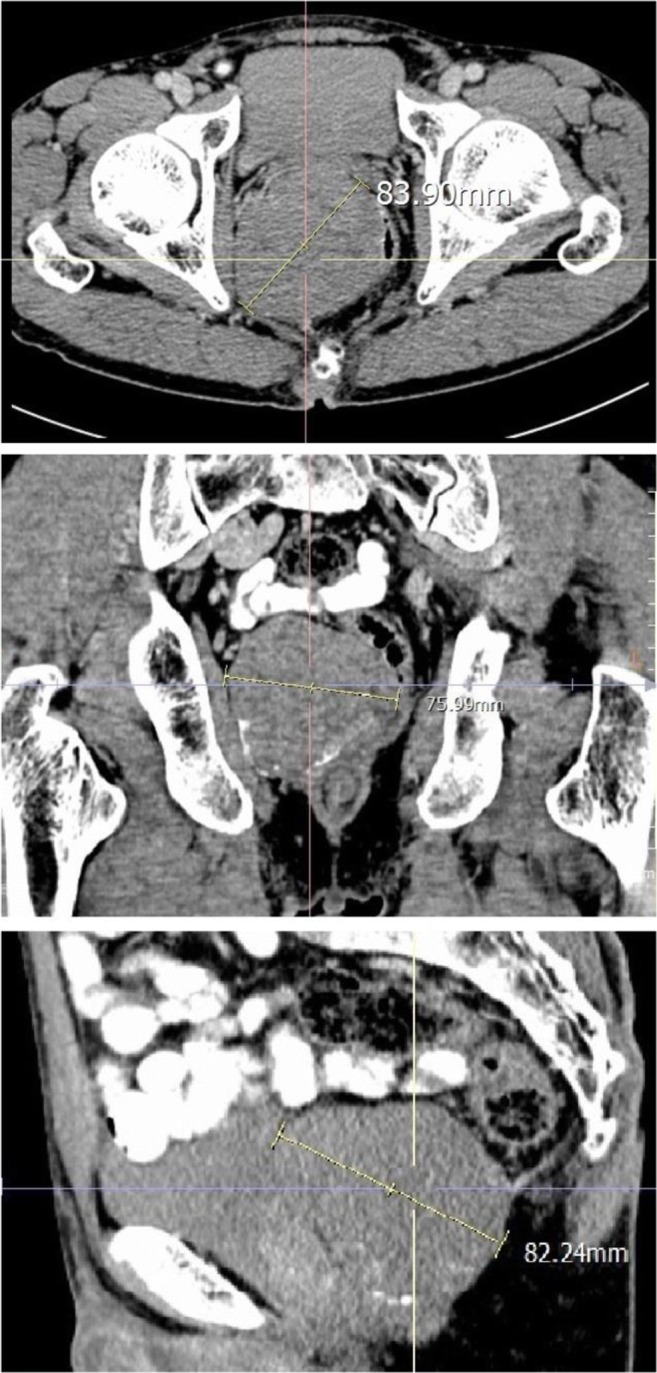
Comparison of axial, coronal, and sagittal images of a pelvic tumor with sample measurements. The maximum value was selected to determine the long diameter (LD)

**Fig. 3:**
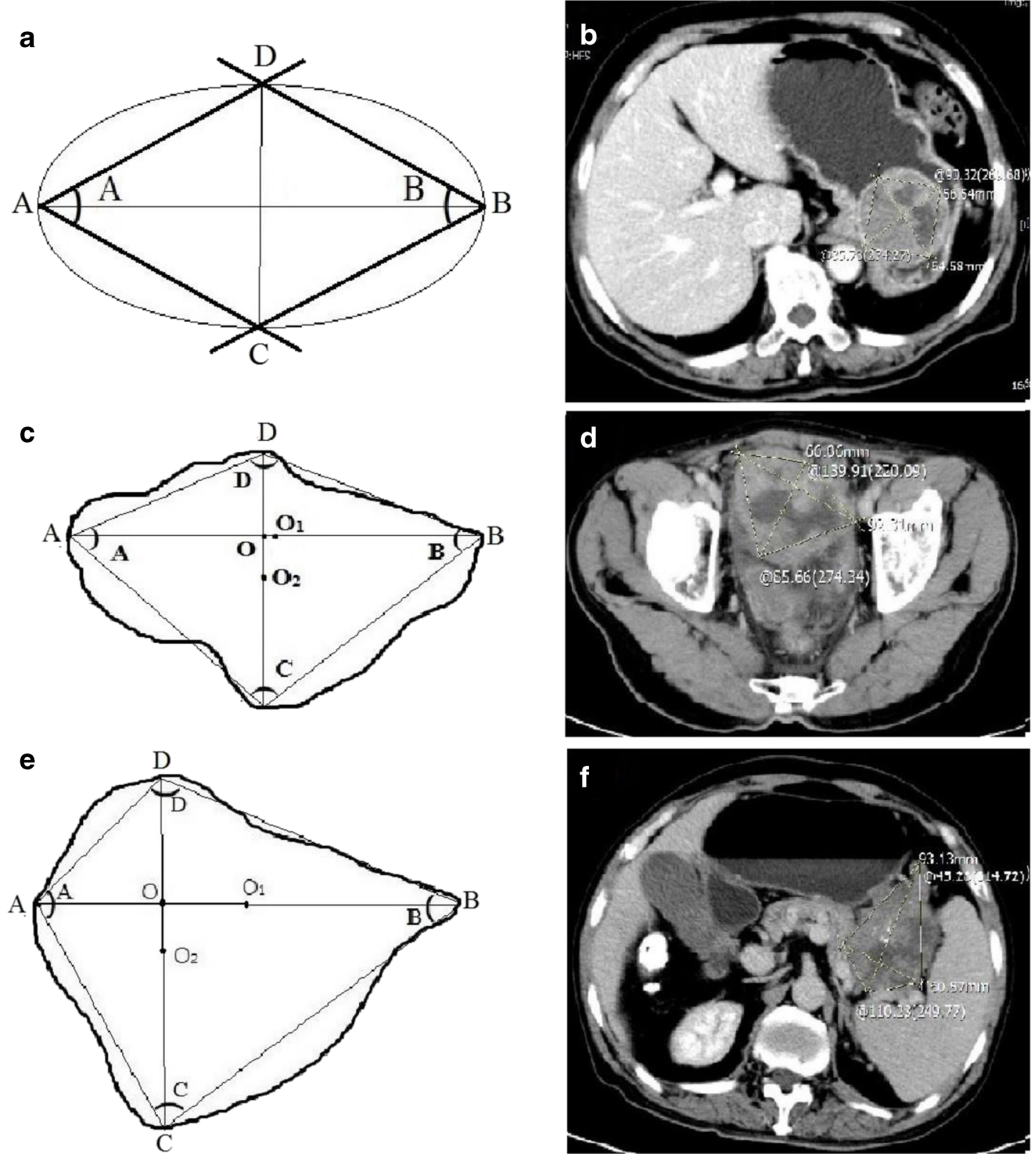
Two-dimensional schematic diagram and actual tumor measurement picture of three types of GISTs with different shapes. **a**, **b** For the regular oval or circular lesions, we took angle A (90.32′) and angle B (85.73′) as the big angle (BiA) and the small angle (SmA). Then, we obtained the BiA/SmA ratio (90.32′/85.73′), which was approximately 1.05. Then, the tumor was classified as intermediate risk or below, and it was pathologically classified as an intermediate-risk GIST. **c**–**f**) When the lesions were irregular, the BiA/SmA ratios were angle A/angle B (139.91′/65.66′ = 2.13) and angle D/angle C (110.23′/45.10′ = 2.44), respectively. Both tumors were classified as high-risk GISTs, which was consistent with the pathological risk classification

**Fig. 4:**
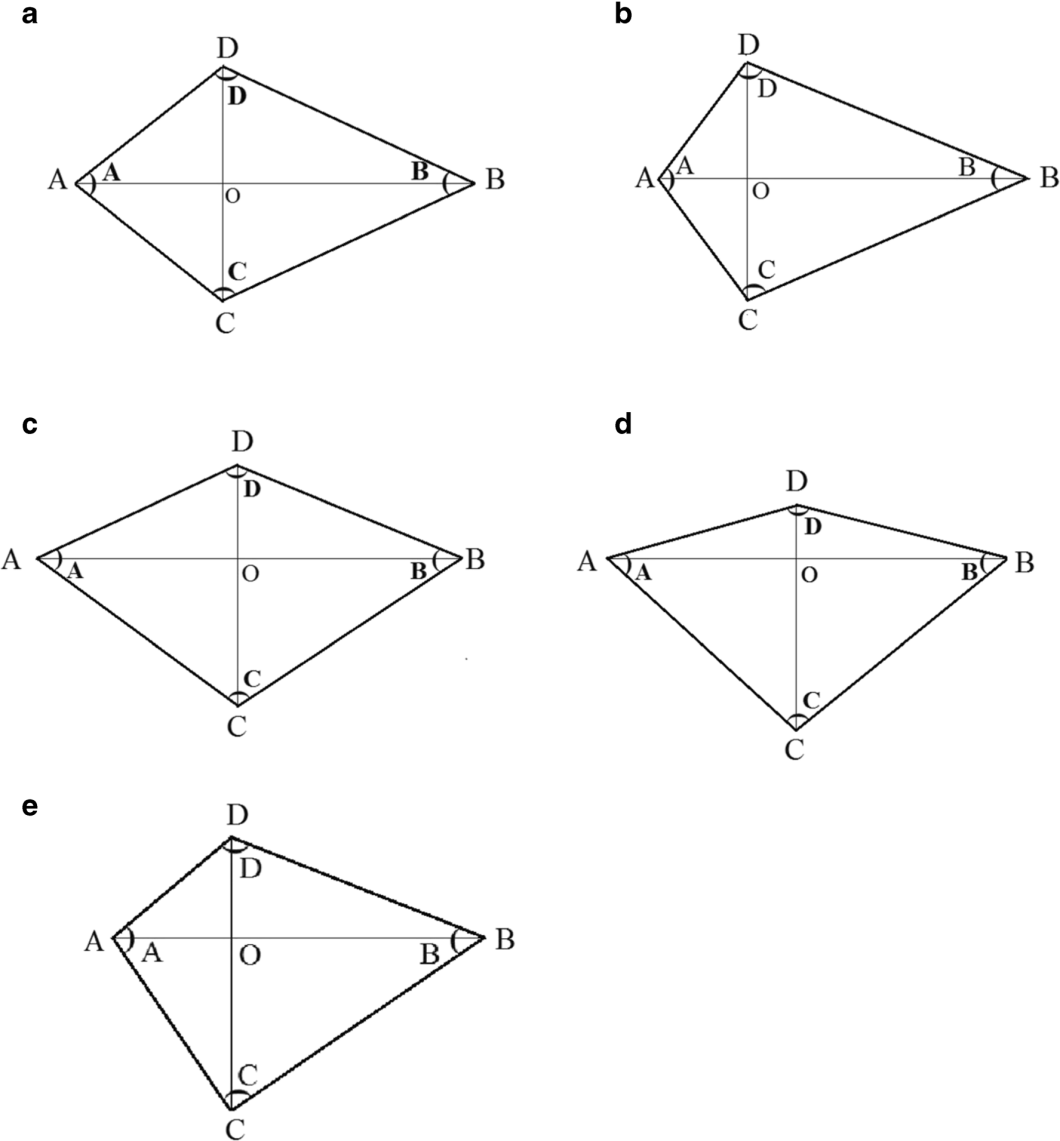
For understanding, we isolate the LD and SD of the irregular lesion for analysis. **a**, **b** Angle A/angle B ratio is larger than angle D/angle C ratio visually, and angle D/angle C ratio is close to 1. When point “A” is closer to the point “O”, the value of angle A/angle B ratio will be larger, and the lesion grows toward the “OB” direction as a whole, and the lesion is more irregular as a whole. According to the results of our analysis, the risk stratification of the lesion will be higher. The explanations of (**c**) and (**d**) are the same as (**a**) and (**b**). **e** When it is difficult to judge the values of angle A/angle B ratio and angle D/angle C ratio, we will compare the two ratios and select the larger ratio for analysis

The largest dimension of the lesion was selected when measuring the LD and SD of the lesion in the cross-sectional, coronal-reconstructed imaging, or sagittal reconstructed imaging (multiplanar reformation (MPR) imaging). Comparing the size of the cross-sectional, coronal, and sagittal planes of the tumor, the LD (line “AB”) (Fig. 2) of the lesions was determined according to the Response Evaluation Criteria in Solid tumors [RECIST] [9], and then the maximum SD (line “CD”) perpendicular to the LD was measured in this section. The four angles of the quadrangle ABCD were measured and each angle named by its summit (A, B, C, D). **A**ngle measurements on the PACS system were very convenient, with three points determining an angle. The angle corresponding to the LD was angle A and angle B (A ≥ B), and the angle corresponding to the SD was angle C and angle D (D ≥ C). Line “AB” and line “CD” intersected at point “O.” Point “O_1_” and point “O_2_” were the middle of line “AB” and line “CD.” When the mass was regular, oval, or circular, we took the two opposite angles of the LD, angle A and angle B (Fig. 3a, b). For irregular lesions, if it was easy to discern the larger ratio of the angle A/angle B ratio to angle D/angle C ratio, we can directly measure the opposite angles corresponding to the larger ratio (Fig. 3c, d). If it was difficult to discern the two ratios, we would compare the angle A/angle B ratio with the angle D/angle C ratio and select the larger ratio for analysis (Fig. 3e, f). We used the pattern diagram to explain the irregular lesions in detail (Fig. 4). We took out the LD and SD of the lesion separately. In Fig. 4a, b, the LD and the SD intersected at point “O.” When the LD crossed the midpoint of the SD or was within its vicinity, angle D was approximately equal to angle C. The closer point “A” was to point “O,” the larger the angle A was. The larger angle A/angle B ratio was, the mass would grow more prominently in the “OB” direction, and then the more irregular the whole lump was. The principles of Fig. 4c, d are the same as Fig. 4a, b. If it was difficult to judge the size of the angle A/angle B ratio and the angle D/angle C ratio visually (Fig. 4e), we measured each angle and calculated the angle ratio and then compared the two ratios and selected a larger ratio for analysis. Two of the authors (X and W) independently measured the LD and SD, BiA, and the opposite SmA of each lesion, and the LD/SD ratio and BiA/SmA ratio were calculated. For the length, angle, and CT attenuation coefficient to be measured, when the difference between the two radiologists was within 0.1 cm, 1′ and 1 HU, respectively, the average was taken. If this range was exceeded, the two authors would negotiate together to find out the cause of the measurement error and re-measured to determine the final result. The values of measured LD, SD, LD/SD ratios; BiA, SmA, and BiA/SmA ratios; and UE, ApU, VpU, and DpU were used in the analysis.

### Statistical analysis

In our study, we divided the mitotic values into ≤ 5 and > 5 per 50 HPF (high-power field) two levels, dividing the risk classification into very low- or low-, intermediate-, and high-risk three levels. To analyze whether there were significant differences between risk grading and CT features, BiA, BiA/SmA ratio, LD, or LD/SD ratio, we used the chi-square test for the categorical variables and the *t* test or nonparametric test for the continuous variables. A *p* value of less than 0.05 was considered to indicate a significant difference. Receiver operating characteristic (ROC) analysis was performed to determine the optimal cutoff value of the BiA, BiA/SmA ratio, LD, and LD/SD ratio differentiating the high-risk GISTs and intermediate-risk grade or differentiating the mitosis values ≤ 5 and > 5/50 HPF. The correct index, also known as the Youden index, was the sum of sensitivity and specificity minus 1. In the Excel table, the results of the subtraction were sorted to obtain the maximum value of the correct index, which was the optimal cutoff. Then, the maximum sum of specificity and sensitivity was obtained. Hierarchical logistic regression analysis was used to identify independent influencing factors affecting the risk grading of GISTs, using binary logistic regression analysis to identify independent influencing factors affecting the mitosis values. In addition, some data groupings would be reasonably merged in the analysis because of the need.

## Results

**Fig. 5:**
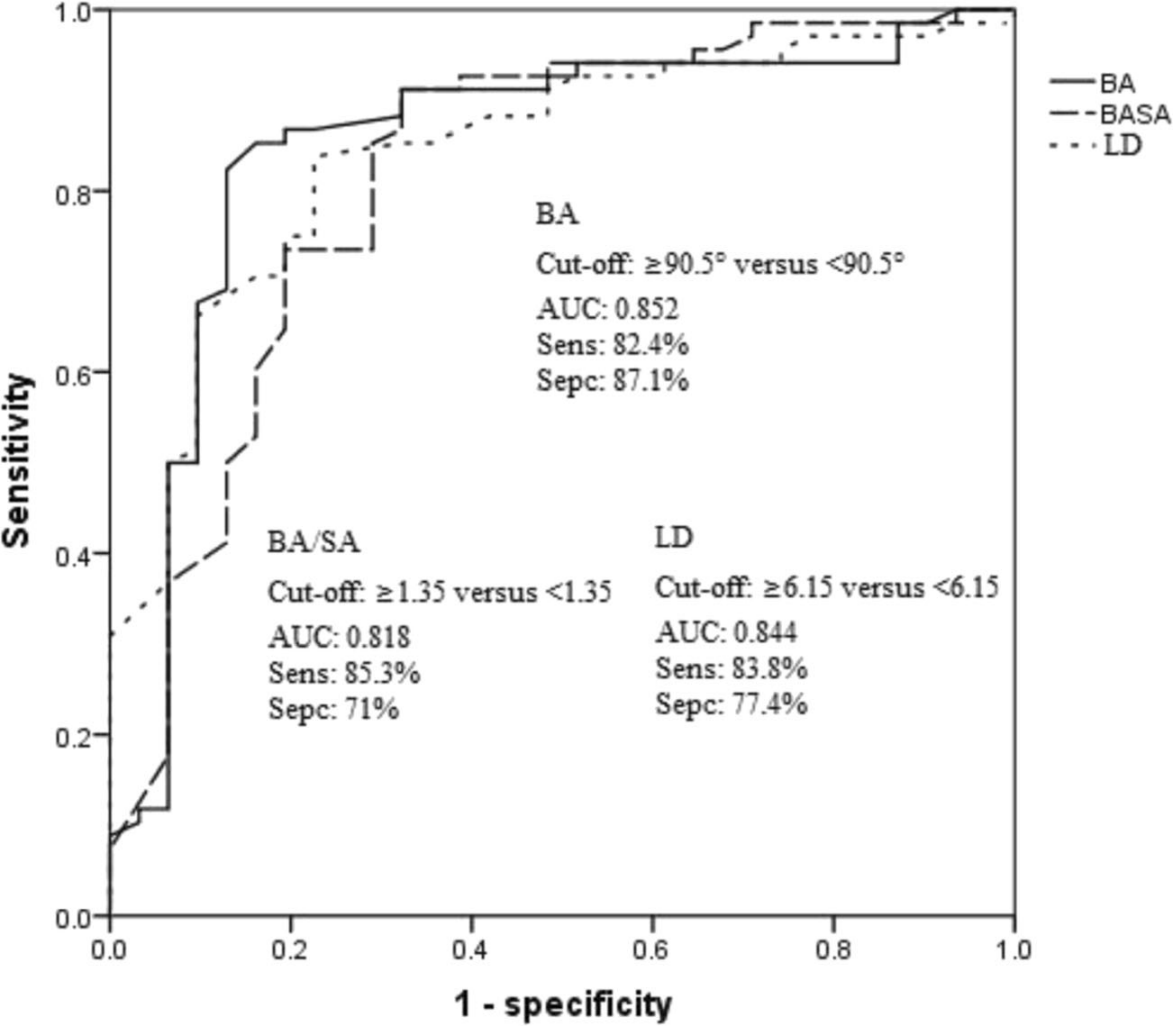
Receiver operating characteristic curve of the LD, BiA, and BiA/SmA ratio for identifying high-risk GISTs. When we set the cutoff value for BiA as 90.5°, BiA/SmA ratio as 1.35, and long diameter (LD) as 6.15 cm, the sensitivity values for high-risk GISTs were 82.4%, 85.3%, and 83.8%, respectively; the specificity values were 87.1%, 71%, and 77.4%, respectively; and the AUC values were 0.852, 0.818, and 0.844, respectively. AUC, area under the curve; Sens, sensitivity; Spec, specificity

**Fig. 6:**
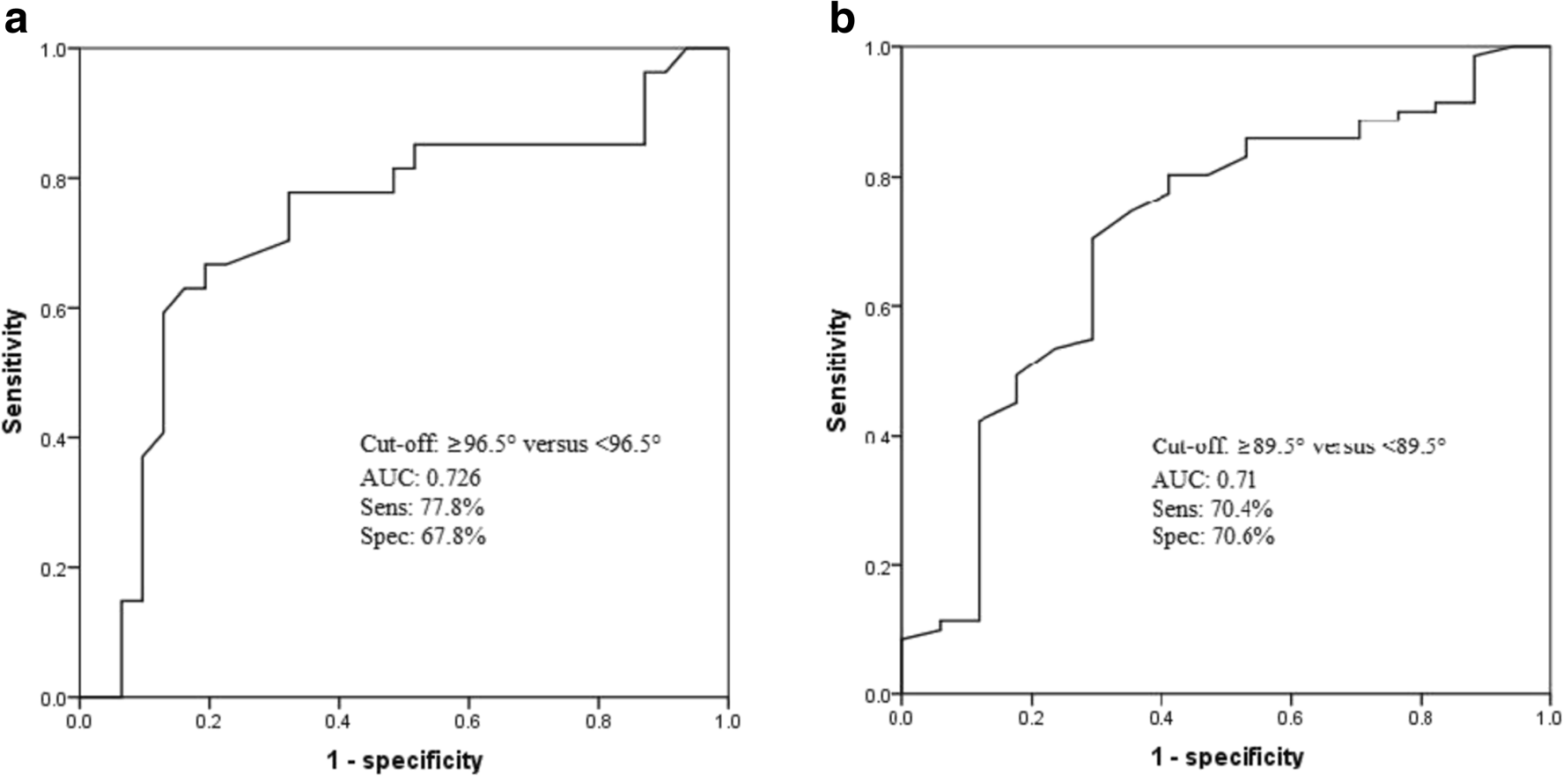
**a** When we set the cutoff value for BiA at 96.5°, the sensitivity and specificity for distinguishing high-risk from intermediate-risk GISTs were 77.8% and 67.8%, respectively, and the AUC value was 0.726. **b** When we set the cutoff value for BiA at 89.5°, the sensitivity and specificity for mitotic counts > 5/50 HPF were 70.4% and 70.6%, respectively, and the AUC was 0.71. AUC, area under the curve; Sens, sensitivity; Spec specificity

The relationship between qualitative and quantitative CT findings was presented in Tables 1 and 2. The ROC curves for LD, BiA, and BiA/SmA were shown in Fig. 5. It included the best cutoff value for distinguishing high-risk and other risks of GISTs and their sensitivity, specificity, and the area under curve (AUC) values. In addition, LD could not effectively distinguish between intermediate-risk and high-risk GISTs (*p* = 0.057), but BiA could distinguish them (*p* < 0.05). The ROC curve for BiA to distinguish high-risk from intermediate-risk GISTs was shown in Fig. 6a. For GISTs, the LD, location, and BiA/SmA ratio were the independent influencing factors affecting the risk classification by hierarchical logistic regression analysis, and the *p* values were 0.005 (95%CI = 1.381~7.737), 0.006 (95%CI = − 51.103~− 9.156), and 0.019 (95%CI = 3.349~37.079), respectively. In the bivariate correlation analysis, the Spearman correlation coefficients of LD with BiA and BiA/SmA ratio were 0.586 and 0.622, respectively (*p* < 0.001, respectively), indicating that LD was moderately positively correlated with BiA and BiA/SmA ratio. Although the BiA and BiA/SmA ratio had some correlation with LD, there were still many lesions with small size but very irregular, and some lesions were large but very regular.

**Table 1:** Relationship between risk grading and sex, qualitative CT findings or Ki-67 index

	Risk grading			*Χ*^2^ value	*p* value
	VL and L	Intermediate	High		
Sex				1.816	0.403
Men	19 (46.3%)	11 (40.7%)	19 (57.6%)		
Women	22 (53.7%)	16 (59.3%)	14 (42.4%)		
Growth^b^ pattern				20.318^a^	< 0.001
Endoluminal	21 (52.5%)	10 (37%)	2 (6.3%)		
Exophytic	12 (30%)	13 (48.1%)	28 (87.5%)		
Mixed	7 (17.5%)	4 (14.8%)	2 (6.3%)		
Calcification				6.606	0.037
Absent	38 (92.7%)	21 (77.8%)	23 (69.7%)		
Present	3 (7.3%)	6 (22.2%)	10 (30.3%)		
Necrosis				19.444	< 0.001
Absent	35 (85.4%)	12 (44.4%)	13 (39.4%)		
Present	6 (14.6%)	15 (55.6%)	20 (60.6%)		
Shape^c^				38.208	< 0.001
Regular	37 (90.2%)	13 (48.1%)	6 (18.8%)		
Irregular	4 (9.8%)	14 (51.9%)	26 (81.2%)		
Enhancement pattern				16.191	< 0.001
Heterogeneous	14 (34.1%)	18 (66.7%)	26 (78.8%)		
Homogeneous	27 (65.9%)	9 (33.3%)	7 (21.2%)		
Enhanced peak period^d^				33.726^a^	< 0.001
Arterial phase	2 (4.9%)	1 (3.7%)	17 (53.1%)		
Venous phase	7 (17.1%)	6 (22.2%)	8 (25%)		
Delay period	32 (78%)	20 (25%)	7 (21.9%)		
Ki-67				28.238^a^	< 0.001
≤ 5%	40 (97.6%)	25 (92.6%)	17 (51.5%)		
> 5%	1 (2.4%)	2 (8.4%)	16 (48.5%)		

*VL*, very low-risk; *L*, low-risk

^a^Kruskal–Wallis test

^b^One case was diffuse growth and the other was multiple gastric GISTs

^c^In a case of multiple gastric GISTs, one grew regularly and one grew irregularly

^d^One lesion had no delay period

**Table 2:** Relationship between risk grading and age or quantitative CT findings

	Risk grading			*p* value
	VL and L	Intermediate	High	
Age (year)	57.6 ± 10.9	57.9 ± 7.4	55.4 ± 8.5	0.198^a^
LD (cm)	3.0 ± 1.3	5.9 ± 1.9	6.9 ± 1.8	< 0.001^b^
LD/SD	1.25 ± 0.15	1.28 ± 0.17	1.32 ± 0.26	0.786^a^
BiA (′)	78 ± 7	95 ± 16	108 ± 18	< 0.001^a^
BiA/SmA	1.07 ± 0.07	1.32 ± 0.31	1.62 ± 0.52	< 0.001^a^
plain scan (HU)	36 ± 6	32 ± 7	36 ± 6	0.056^a^
Ap (HU)	16 ± 12	15 ± 12	36 ± 24	< 0.001^a^
Vp (HU)	27 ± 16	26 ± 15	35 ± 16	0.056^a^
Dp (HU)	28 ± 12	32 ± 16	32 ± 13	0.228^a^

^a^Kruskal-Wallis test

^b^One-way ANOVA

Five CT features or pathological results were significantly suggestive of mitotic value > 5 per 50 HPF rather than < 5 per 50 HPF: exophytic growth pattern (*p* = 0.015), irregular shape (*p* = 0.001), bigger BiA (*p* = 0.007), ApU (*p* = 0.008), and Ki67 index (> 5%) (*p* < 0.001), However, shape and Ki-67 index were independent factors of the mitotic value by binary logistic regression analysis, and the *p* values were 0.036 (95%CI = 1.117~26.123) and less than 0.001 (95%CI = 2.857~40.654) respectively. The regular shape or lower Ki-67 index indicated lower mitotic counts, and the accuracy of prediction was 87.8%. When we set the cutoff value for the BiA at 89.5°, the sensitivity and specificity for mitotic value > 5/50 HPF were 70.4% and 70.6%, respectively, and the AUC was 0.71 (Fig. 6b)

## Discussion

The method for quantifying tumor shape can predict the risk level and mitotic value of GISTs, especially for high-risk grading and mitotic value > 5/50 HPF. BiA has better predictive efficacy than LD in distinguishing between intermediate-risk and high-risk GISTs or high-risk and other risks GISTs.

Enhancement CT is the standard method for GIST imaging [10] and plays an important role in the preoperative evaluation of GISTs [6, 11–13]. Some measuring tools of PACS [14, 15] could facilitate the diagnosis and risk assessment of GISTs. Computer-extracted shape cues could be used to distinguish radiographically similar pathologies of adenocarcinomas from granulomas on the lung [16]. Zanoni et al [17] found that shape may be a variant source of the tumor. For a solid tumor to grow in a confined space defined by the surrounding tissue, it must overcome the resulting compressive forces. Cheng et al [18] found a strong correlation between the peri-spheroid solid stress distribution and spheroid shape, a result of the suppression of cell proliferation and induction of apoptotic cell death in regions of high mechanical stress. In addition, Mazurowski et al [19] reported that shape features were strongly associated with genomic subtypes and patient outcomes in lower-grade glioma, and Okabe et al [8] reported that irregular tumor shape on preoperative computed tomography was a negative prognostic factor after curative surgery for pancreatic neuroendocrine tumors. Therefore, we believe that there is a significant correlation between the shape of GIST and risk level. Most studies divided tumors into regular and irregular shapes [3, 5], but in our study, we quantified the tumor shape, transforming qualitative analysis into quantitative analysis on PACS by CT three-dimensional reconstruction imaging, which, to our knowledge, has not been previously reported. According to the improved classification of GISTs proposed by the NIH in 2008, lesion size, location, and rupture were used as independent factors in predicting the risk grading in the preoperative CT findings [2]. In this study, the BiA/SmA ratio is verified as an independent influencing factor affecting the risk classification. We also find that the BiA or BiA/SmA ratio has a better effect on predicting grading of GISTs less than 10 cm than LD, especially in distinguishing between intermediate-risk and high-risk GISTs or high-risk and other risk GISTs. In addition, quantifying the shape to study the risk grading of GISTs is the highlight of this research.

Tumor mitotic figure is one of the independent prognostic factors [2, 20, 21]. Ki-67 is a proliferation marker for tumors and has been used for tumor staging, poorly differentiated malignancies [22]. Li et al [23] reported that the Ki-67 index was positively correlated with mitotic counts. Meanwhile, Kemmerling et al [24] reported that Ki-67 could accurately predict mitotic counts. Our research was consistent with them. In our study, GISTs with mitotic value > 5/50 HPF more commonly had an exophytic growth pattern, irregular shape, bigger BiA and Ap, and Ki67 index (> 5%), but only shape and Ki-67 index were independent factors of mitotic value by hierarchical logistic regression analyses. This finding was different from that in a previous study [25], in which size was the only significant predictor of high mitotic counts. However, there was no significant difference between the mitotic value and different sizes (*p* = 0.075) in our research, and our research was very similar to that of Chen et al [26], in which there was no difference between mitotic counts and size, while the difference between mitotic counts and shape or growth patterns was statistically significant. In addition, some studies evaluated tumor response to treatment with CT attenuation coefficient on enhanced CT [27, 28], but they did not study the relationship between triphasic CT attenuation coefficients and mitotic value. In our study, the absolute CT attenuation coefficient of the arterial phase (ApU) could predict the mitotic value to a certain extent, and the lesion with a higher ApU value was more likely to have a higher mitotic value. The higher the ApU value was, the more abundant the blood supply of the tumor was. Therefore, it is suspected that the mitotic value may be related to the blood supply mode of GISTs, but the relationship between mitotic value and Ap still needs further research.

There were several limitations in our present study. First, the evaluation of cases was retrospective, making selection bias unavoidable. Second, all patients did not undergo a uniform pre-examination of gastrointestinal preparation, as some patients used warm water, and some used oral gastrointestinal contrast agents, which would have some impacts on the results. Third, the number of cases in the retrospective study remained small, and some of the data subgroups had to be combined to reach statistically relevant class size.

In conclusion, the method for quantifying the tumor shape can predict the risk level and mitotic value of GISTs, especially for high-risk grading and mitotic value > 5/50 HPF. BiA has better predictive efficacy than LD in distinguishing between intermediate-risk and high-risk GISTs or high-risk and other risk GISTs.

## Abbreviations

Ap: Arterial phase
AUC: The area under the receiver operating characteristic curve
BiA: Big angle
CT: Computed tomography
GISTs: Gastrointestinal stromal tumors
FPF: High-power field
LD: Long diameter
ROC: Receiver operating characteristic
SD: Short diameter
SmA: Small angle
UE: Unenhanced
